# Cannabinoid Receptor 2-Mediated Attenuation of CXCR4-Tropic HIV Infection in Primary CD4+ T Cells

**DOI:** 10.1371/journal.pone.0033961

**Published:** 2012-03-20

**Authors:** Cristina Maria Costantino, Achla Gupta, Alice W. Yewdall, Benjamin M. Dale, Lakshmi A. Devi, Benjamin K. Chen

**Affiliations:** 1 Department of Infectious Diseases, Department of Medicine, Immunology Institute, Mount Sinai School of Medicine, New York, New York, United States of America; 2 Department of Pharmacology and Systems Therapeutics, Mount Sinai School of Medicine, New York, New York, United States of America; George Mason University, United States of America

## Abstract

Agents that activate cannabinoid receptor pathways have been tested as treatments for cachexia, nausea or neuropathic pain in HIV-1/AIDS patients. The cannabinoid receptors (CB_1_R and CB_2_R) and the HIV-1 co-receptors, CCR5 and CXCR4, all signal via Gαi-coupled pathways. We hypothesized that drugs targeting cannabinoid receptors modulate chemokine co-receptor function and regulate HIV-1 infectivity. We found that agonism of CB_2_R, but not CB_1_R, reduced infection in primary CD4+ T cells following cell-free and cell-to-cell transmission of CXCR4-tropic virus. As this change in viral permissiveness was most pronounced in unstimulated T cells, we investigated the effect of CB_2_R agonism on to CXCR4-induced signaling following binding of chemokine or virus to the co-receptor. We found that CB_2_R agonism decreased CXCR4-activation mediated G-protein activity and MAPK phosphorylation. Furthermore, CB_2_R agonism altered the cytoskeletal architecture of resting CD4+ T cells by decreasing F-actin levels. Our findings suggest that CB_2_R activation in CD4+ T cells can inhibit actin reorganization and impair productive infection following cell-free or cell-associated viral acquisition of CXCR4-tropic HIV-1 in resting cells. Therefore, the clinical use of CB_2_R agonists in the treatment of AIDS symptoms may also exert beneficial adjunctive antiviral effects against CXCR4-tropic viruses in late stages of HIV-1 infection.

## Introduction

Cannabinoid agonists are currently under investigation for the treatment of AIDS-associated cachexia, nausea, and neuropathic pain [1]–[3]. One such drug, dronabinol (Δ^9^-THC; Marinol®), has won Food and Drug Administration (FDA) approval for treatment of HIV-associated anorexia [4]. Additionally, the prescription of smoked or ingested cannabis (marijuana) for treatment of AIDS-related symptoms has been approved in 14 states [5]. Despite the use of cannabinoids by HIV/AIDS patients, few studies have investigated the impact of such drugs in regard to viral pathogenesis or immune regulation. Early studies conducted in the pre-HAART era suggested a positive correlation between development of opportunistic infection, progression to AIDS, and marijuana use [6], [7]. Yet recent analysis of HIV/AIDS patients enrolled a randomized, placebo-controlled clinical trial designed to study the outcome of cannabinoid administration have indicated that cannabinoid use does not result in significant immunosuppression [1]. Indeed, both smoked marijuana and dronabinol were reported to increase total CD4+ T cell number [1] and naïve T cell number [8] over a 21-day period. A decrease in viral load was also observed in these patients [1]. Similarly, in SIV infected rhesus macaques, Δ^9^-THC exposure reduced viral load and CD4+ T cell depletion, significantly increasing animal survival over an 11 month period [9]. Despite these findings, the mechanisms by which cannabinoid drugs can influence viral replication or pathogenicity remain unknown.

Cannabinoid agonists activate the CB_1_R and CB_2_R cannabinoid receptors. Like the HIV chemokine co-receptors CXCR4 and CCR5, CB_1_R and CB_2_R are members of the Gαi-coupled family A GPCRs [10]. CB_2_R is highly expressed on all CD4+ T cells [11], whereas CB_1_ expression is found in activated, memory subsets [12]. CB_1_ and CB_2_ have been classified as immunosuppressive receptors on CD4+ T cells [13], although antagonism of CB_1_R and CB_2_R does not enhance immune activation and knock-out mice do not exhibit differences in T cell frequency or increases in autoimmune pathogenesis [14]. The mechanism(s) by which cannabinoid agonists can modulate CD4+ T cell function remain unclear. Activation of CB_2_R has been shown to inhibit inflammatory cytokine production in CD4+ T cells [11], which may account for the decrease in autoimmune pathogenesis observed in therapeutic trials of cannabinoid agonists in animal models of multiple sclerosis [14], [15]. CB_2_R may also function as a chemotactic modulator, as CB_2_R activation inhibits CXCR4-induced chemotaxis in transformed lymphocytes [16]. CB_2_R has further been shown to regulate lymphocyte egress from the bone marrow in a role previously attributed largely to CXCR4 [17], [18]. These findings suggest that CB_2_R may play a role in regulating chemokine receptor signaling, specifically the activity of CXCR4. Such cross-talk between CB_2_R and CXCR4 may have implications for AIDS patients who take cannabinoid-derived agents for therapeutic purposes.

Although coreceptor signaling is not essential for HIV-1 infection, several recent studies have suggested that chemokine receptor signaling enhances infection of resting CD4+ T cells [19]–[21]. These cells express CXCR4, but not CCR5, whose expression is restricted to a small subset of memory CD4+ T cells [22]. In patients, the emergence of CXCR4-tropic virus usually occurs after years of infection and correlates with more rapid progression to AIDS [23], [24], [25]. Viral conversion to CXCR4-tropism increases the number of targets available to the virus [26]. Additionally, as HIV-1 can establish latency in resting T cells [27], a switch to CXCR4-tropism could enhance the establishment of a latent pools of virus within lymphoid tissues. The increased number of new targets may explain the rapid decline in CD4+ T cell numbers and increased viral load in late-stage AIDS patients with CXCR4-tropic virus [1], [24]. The late-stage patients who frequently harbor CXCR4-tropic virus are also the most likely to benefit from cannabinoid drug use. It is therefore relevant to study the potential for cannabinoid signaling to modulate CXCR4 activity and alter the course of HIV infection, Interactions between GPCRs like CB_2_R and CXCR4 can cause cross-desensitization, allosteric modulation, dimerization, changes in receptor localization, and alteration of physiological function among GPCR pairings [28]. Given that direct antagonism of chemokine receptor function can block viral infection [19], [29], [30], it is possible that allosteric modulation of CXCR4 through a GPCR partner may also reduce HIV-1 permissiveness. Indeed, oligomerization of the chemokine co-receptors including CXCR4 using conformationally specific monoclonal antibodies can inhibit HIV-1 entry into target cells [31], [32]. These experiments demonstrate signaling-independent modulation of coreceptor function. Allosteric agents that disrupt HIV-1 infection by modulating chemokine receptor signaling have not yet been identified. Should CB_2_R-induced signaling alter CXCR4 co-receptor function, this would represent first known example of a signaling-dependent GPCR interaction leading to viral inhibition.

To test this hypothesis, we examined the effect of cannabinoid receptor activation on HIV viral transmission and productive infection in CD4+ T cells using a GFP-expressing, CXCR4-tropic HIV-1 variant. We found that activation of CB_2_R on CD4+ T cells significantly inhibited viral infection in a CB_2_R -selective and dose-dependent manner. Viral inhibition was more pronounced in resting cells that were activated after infection. We investigated signaling in these cells and found that CB_2_R agonism significantly decreased SDF-1α-induced CXCR4 activation. Furthermore, CB_2_R agonism altered HIV-induced cytoskeletal rearrangement, associated with productive HIV infection in resting cells. We found that CB_2_R was not sufficient to block viral transfer or fusion, but did significantly diminish productive viral infection. We conclude that CB_2_R is a novel modulator of CXCR4-tropic HIV infection in CD4+ T cells.

## Materials and Methods

### Cell culture reagents

Anonymous blood donations were obtained from New York Blood Center. Cells were cultured as previously reported [33]. The purified monoclonal antibodies (mAbs) 〈CD2 (OKT3) and 〈CD28 (28.2) were purchased from eBioscience. Phytohemagglutinin (PHA) and carboxyfluorescein succinimidyl ester (CFSE) was purchased from Sigma. Recombinant human IL-2 and CXCR4 antagonist AMD3100 were obtained through the AIDS Research and Reference Reagent Program (NIH). Fluorophore-conjugated CXCR4 (12GS), CCR5 (T21/8), CD25 (BC96), and CD45RO were purchased from Biolegend. The CB2 agonists JWH-133, JWH-150, Ser160, 2-arachidonoylglycerol, and anandamide and the CB2 antagonist AM630 were purchased from Tocris.

### Cell purification and sorting

Total CD4+ T cells were isolated from healthy HIV/hepatitis B virus-seronegative donors as described previously [33]. For FACS-sorting, CD4+ T cells were labeled with Live/Dead for viability (Invitrogen), stained for CD45RO, and sorted with a FACS Aria (BD Biosciences).

### Viral constructs

HIV NL-GI and Gag-iCherry are NL4-3 based CXCR4-tropic HIV-1 molecular clones that have been described previously [34]. NL-GI expresses green fluorescent protein (GFP) in place of the viral early gene nef, and nef expression is maintained by insertion of an internal ribosome entry site (IRES) [35]. Gag-iCherry carries the GFP variant Cherry inserted internally into Gag between the MA and CA domains. For CCR5-tropic virus, a variant of NL-GI expressing the *Env* gene from the molecular clone JRFL [36] was used. Virus was produced in HEK293T cells and p24 concentration was calculated by ELISA prior to use.

### Cell-Free Infection Assay

CD4+ T cells were thawed, resuspended in RPMI medium containing 20 U/ml recombinant IL-2 and stimulated with 1 µg/ml PHA (Sigma) overnight. Cells were cultured for four days and reseeded into 96 well flat-bottom plates (Costar) at a density of 10^5^ cells/well prior to treatment and infection. Treated cells were incubated with antagonist or vehicle (DMSO) for one hour at 37°C followed by incubation with agonist or vehicle (DMSO or 0.1% ethanol) for another three hours. Following this treatment, triplicate cultures were infected with 10 ng/well HIV NL-GI. To assess HIV infection, fluorescence was assessed at day 4 post-treatment. Harvested cells were stained for viability and fixed with 4% paraformaldehyde, prior to acquisition on a FACSCalibur (BD Biosciences) and analysis with FloJo software (TreeStar).

### Cell-Associated Infection Assay

HIV-expressing Jurkat donor cells were generated by transfection using HIV Gag-iCherry, as described previously [34]. To generate infected CD4+ T cell donors, PHA activated CD4+ T cells were spinoculated with either HIV NL-GI or for 90 minutes at 1200×g. After 24–48 hours, approximately 10–30% of donor cells were infected. Donor cells were labeled with 10 µM CellTracker blue (CMF2HC) fluorescent dye (Invitrogen) and then co-cultured with unstimulated and antagonist and/or agonist-treated target cells in a 1∶1 ratio for 3 hours at 37°C, as described previously [33]. Virus transfer was terminated by washing with PBS and treatment with 0.05% trypsin-EDTA (Invitrogen) for 5 minutes. Cells were stained for viability and for CD45RO prior to fixation and acquisition on a LSRII (BD Biosciences). Sorted GFP^−^Cherry^−^ CD45RO^+^ or CD45RO^−^ targets were seeded into 96-well plates coated with 2.5 µg/ml anti-CD3 in RPMI media containing 1 µg/ml anti-CD28 and 20 U/ml rIL-2 for activation. After 4 days, cells were harvested, fixed, and analyzed for fluorescence.

### Quantitation of Viral Membrane Fusion

Cell-free viral fusion was measured using a method described previously [37]. BlaM-VPR was a gift from Michael Miller (Merck Research Laboratories). Viral infections were done with 20 ng of virus at a concentration of 200 ng/ml for 2 hrs at 37°C. Cells were analyzed on an LSRII flow cytometer (Becton Dickinson).

### Signaling Studies

For GTPγS binding, JWH-133 treated CD4+ T cells were permeabilized with CHAPS and incubated with increasing concentrations of SDF-1α. Incorporation of radiolabelled GTPγS was assayed as described [38]. For kinase phosphorylation and western blotting, primary CD4+ T cells were treated with antagonist and/or agonist in serum-free RPMI for a total of 4 hours prior to treatment with SDF-1α (PeproTech) or NL4.3 HIV at various times indicated. Levels of phosphorylated MAP kinase and total MAP kinase were determined as described [38].

### Data Analysis

Data was analyzed with GraphPad Prism using a paired t-test. Mean and standard error indicate variance between multiple donors, with n as indicated.

## Results

### CB_2_-specific agonists inhibit HIV-1 infection in primary CD4+ T cells

As activation of the cannabinoid receptor CB_2_R has been shown to modify chemokine receptor activity [16] and cannabinoid use in HIV-1 infected individuals is associated with reductions in viral load [1], we hypothesized that CB_2_R agonism may alter the course of HIV-1 infection. Using a GFP-expressing variant of the CXCR4-tropic lab isolate NL4.3 (NL-GI), in which GFP is expressed in place of the viral gene nef [33], we assayed HIV-1 infectivity in primary CD4+ T cells pre-treated with CB_2_R agonist. Purified, blood-derived CD4+ T cells were stimulated with low-dose mitogen for four days prior to a three-hour cannabinoid treatment. To directly test the capacity for CB_2_R activation to inhibit viral infection, we pretreated activated CD4+ T cells with a potent and selective CB_2_R agonist that is approximately 200-fold selective for CB_2_R over CB_1_R (Ki = 3.4 nM) [39] (JWH-133) prior to HIV-1 exposure. Treated cells were then washed and exposed to virus in suspension (10 ng/2×10^5^ cells) for four days. After this time, the frequency of GFP+ infected cells was measured and the frequency of inhibition, as compared to control (DMSO treated) infection, was calculated.

**Figure 1:**
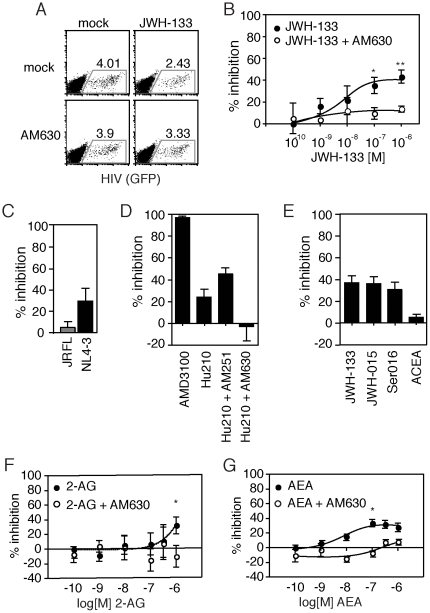
Cannabinoid inhibition of CXCR4-tropic HIV-1 infection is dose-dependent and CB_2_-selective. (A) HIV-1 (GFP) expression 4 days post-infection in primary CD4+ T cells pretreated with 100 nM of the CB2-specific agonist JWH-133 and/or the CB_2_-specific antagonist AM630 in a representative donor. HIV expression (GFP; FL1) is shown on the x-axis, and an empty channel (FL3) is shown on the y-axis. (B) Dose-dependent inhibition of viral infection by JWH-133 in primary CD4+ T cells is blocked by 100 nM of the CB_2_ antagonist AM630 (mean±SEM; n = 7 donors; **p = 0.0032, *p<0.03). (C) Inhibition of CXCR4-tropic (NL4-3) but not CCR5-tropic (JRFL) HIV-1 after 4 days in primary CD4+ T cells pretreated with 100 nM of JWH-133 (mean±SEM; n = 5 donors). (D) Inhibition of viral infection in cells pretreated with the CXCR4 antagonist AMD3100 (which is predicted to block viral binding [57]), or 1 µM of cannabinoid agonists ACEA, Hu210, JWH-133, JWH-015 and Ser016 with or without pretreatment with the CB_1_ antagonist AM251 or the CB_2_ antagonist AM630. (E–F) Dose-dependent inhibition of NL-G1 infection in primary CD4+ T cells pretreated with (F) 2-arachidonoylglycerol (2-AG) or (G) anandamide (AEA) is blocked by 1ìM of the CB_2_ antagonist AM630 (mean±SEM; n = 5 donors; *p<0.03).

When cells were treated with 100 nM of JWH-133 prior to viral exposure, we observed an approximately 40% reduction in HIV-1 infected cells after four days (**Figure 1A–B**). This inhibition was significantly reduced when CB_2_R activity was selectively blocked using the antagonist AM630, indicating that the antiviral activity of JWH-133 is indeed CB_2_R-specific (**Figure 1A–B**). JWH-133-mediated blockade of HIV-1 infectivity was dose-dependent, with an EC50 of 7.59±0.1 nM and a plateau of efficacy at approximately 50% inhibition (**Figure 1B**). Further, the action of this drug was CXCR4-specific, as JWH-133 treatment was not sufficient to inhibit infection with an isogenic virus that carried a CCR5-tropic Env from molecular clone JRFL (**Figure 1C**). Therefore, CB_2_ activation reduces CXCR4-tropic HIV-1 infection in primary CD4+ T cells in a dose-dependent and receptor-specific fashion.

To further confirm this novel role for CB_2_R, we tested other CB_2_R-selective agonists (JWH-015 and Ser016) to see if treatment was sufficient to reduce viral infectivity. Both of the highly specific CB_2_R agonists we tested proved to be antiviral at concentrations of 1 µM; these were JWH-015 (35.9±11.96% inhibition) and Ser016 (30.7±13.72% inhibition) (mean±SEM, n = 8 donors) (**Figure 1D**). Treatment with 1 µM of the pan-cannabinoid agonist Hu210 also significantly reduced HIV-1 infection, although to a lesser extent than with CB_2_R-selective agonists (23.9±7.45%, mean±SEM, n = 8 donors) (**Figure 1D**). The reduction in infection efficiency observed with Hu210 was CB_2_R-specific and was not observed when cells were pretreated with the CB_2_R-selective antagonist AM630 (**Figure 1D**). Consistent with these findings, 1 µM pretreatment with the CB_1_R selective agonist arachidonyl-2′-chloroethylamide (ACEA) did not significantly reduce viral infection (4.76±3.13%, mean±SEM, n = 5 donors) (**Figure 1D**). These results indicate that CB_2_R-selective agonists, but not CB_1_R-selective agonists, can inhibit HIV-1 permissiveness.

Given that the pan-cannabinoid agonist Hu210 possessed antiviral activity, we predicted that naturally occurring endogenous ligands of the cannabinoid receptors, the endocannabinoids 2-arachidonoylglycerol (2-AG) and anandamide (AEA) could reduce infectivity via CB_2_R activation. We found that pretreatment with either 2-AG or AEA significantly inhibited HIV-1 infection in a dose-dependent and CB_2_-specific manner (**Figure 1E–F**). AEA, which is reported to have higher affinity for CB_2_R than 2-AG (Ki = 371 nM versus 1400 nM, respectively) [40], proved to be a more potent inhibitor of virus, with an EC50 of 8.59±0.09 nM, as compared to 1.82±0.25 µM for 2-AG. The antiviral activity of both of these endocannabinoid agents was abrogated by treatment with 1 µM of the CB_2_R-selective antagonist AM630. These data demonstrate that, like synthetic cannabinoid agonists, the endocannabinoids can activate CB_2_R to inhibit HIV-1 infectivity in CD4+ T cells.

### CB_2_ activation does not alter CXCR4 expression or T cell activation

**Figure 2:**
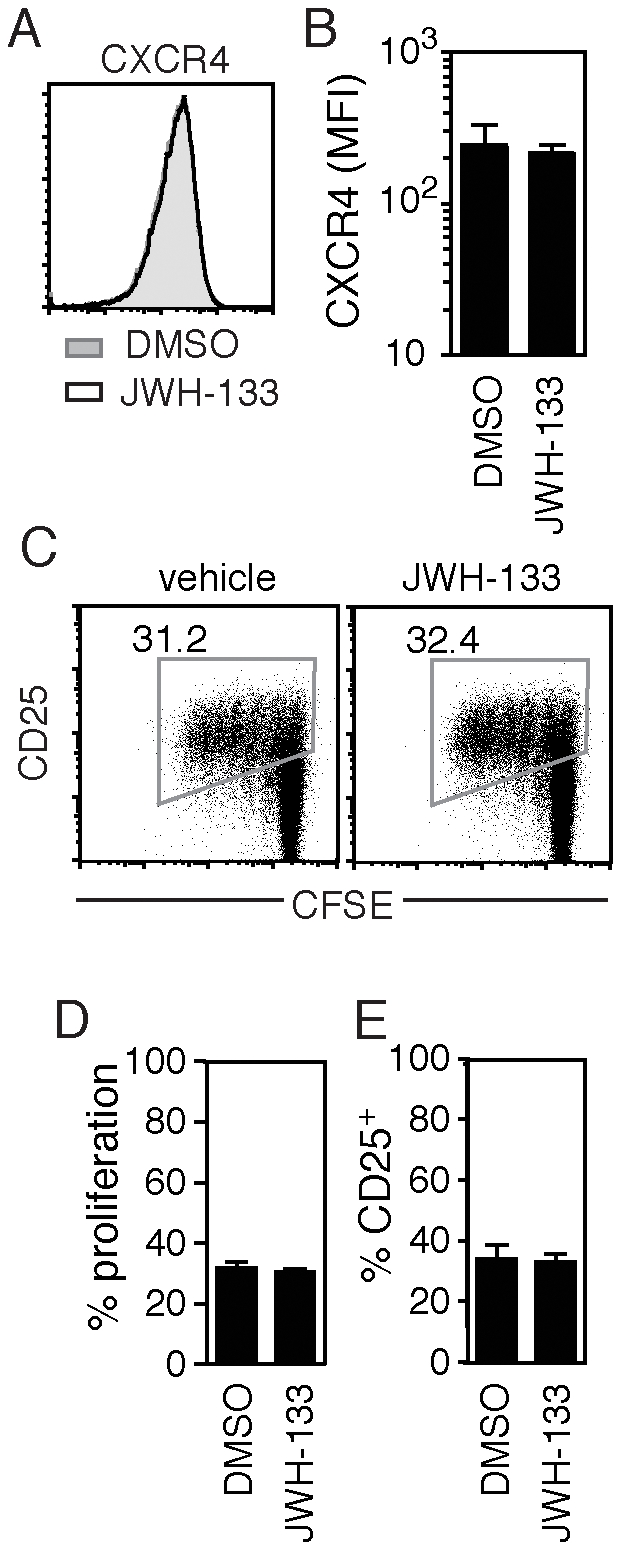
CB_2_ agonism at concentrations sufficient to inhibit HIV-1 infection does not significantly alter cell-surface CXCR4 expression or inhibit cellular proliferation in primary CD4+ T cells. (A–B) Cell surface expression of CXCR4 in CD4+ T cells pretreated with either DMSO or 100 nM of JWH-133 (A) in a representative donor and (B) in multiple donors (mean±SEM; n = 4 donors). (C–E) Proliferation, as indicated by CFSE dilution; and activation, as measured by CD25 expression; in CD4+ T cells, stimulated with cross-linking anti-CD3 and anti-CD28 mAb, 4 days after pretreatment with 100 nM of JWH-133 (C) in a representative donor and (D–E) in multiple donors (mean±SEM; n = 5 donors).

We next sought to determine the mechanism of HIV-1 inhibition via CB_2_R. Previous reports have indicated that cannabinoid treatment of immune cells can lead to changes in cell surface chemokine receptor expression [41], loss of proliferative capacity [11], and reduction in effector function [42], [43]. We examined whether CB_2_R activation in our infection model led to either a reduction in co-receptor expression or in host fitness, rendering the cells incapable of harboring productive viral infection. To test this possibility, we treated CD4+ T cells with the CB2 agonist JWH-133 with a concentration of drug sufficient to inhibit viral infection (100 nM). We found that this treatment did not lead to significant reduction of CXCR4 cell surface expression (**Figure 2A–B**) or total CXCR4 protein expression (data not shown).

Likewise, pretreatment with up to 1 µM of JWH-133 prior to TCR-mediated activation did not reduce T cell activation, as measured by an increase in CD25 expression, or proliferation, as indicated by CFSE dilution after stimulation with anti-CD3 and anti-CD28 antibodies (**Figure 2C–E**). Concentrations of JWH-133 ranging from 1 µM and below did not lead to a change in cell viability as compared to DMSO treated controls (data not shown). Higher concentrations of JWH-133 or Hu210 (10 µM and above) did lead to apoptosis and cell death, consistent with published observations [11]. Our findings indicate that low doses of the CB_2_R agonist JWH-133 are sufficient to inhibit viral infection without significant disruption of CD4+ T cell co-receptor expression, CD4 expression, CD25 upregulation, or proliferation. Further, CB_2_R agonism did not alter the ability of the CD4+ T cell to support viral infection, as JWH-133 treated cells are readily infected by the CCR5-tropic JRFL virus (**Figure 1C**). Taken together, these data suggest that CB_2_R activation is altering a pathway specifically required for CXCR4-tropic infection.

### The antiviral activity of CB2 agonist JWH-133 is enhanced in unstimulated CD4+ T cells

Despite intense study, the importance of GPCR-mediated signaling during CXCR4-tropic HIV-1 infection remains unclear. Recently, several lines of study have demonstrated a role for chemokine receptor signaling in resting CD4+ T cells [44]. Activation of Gαi-coupled chemokine receptors enhances infectivity in resting CD4+ T cells that are stimulated with gp120 or chemokine agonist after exposure to HIV-1 [19], [20]. Conversely, inhibition of GPCR function by pertussis toxin inhibits viral infection [19]. This has been described as a model for latent infection in resting cells [19], [45]. We tested to see if the CB_2_ agonist JWH-133 differentially inhibited HIV-1 infection in resting versus activated cells.

**Figure 3:**
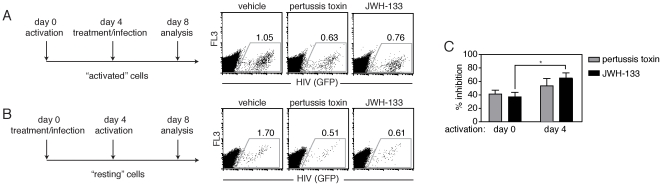
Inhibition of HIV-1 infection by CB_2_ activation is enhanced in CD4+ T cells infected prior to activation. (A–C) Infection, as measured by GFP expression, in primary CD4+ T cells activated with mitogen (A) for 4 days prior or (B) 4 days after treatment with pertussis toxin or 100 nM of the CB2 agonist JWH-133 (A, B) in a representative donor and (C) in multiple donors (mean±SEM; n = 4 donors; *p<0.05).

Cells were activated with mitogen four days before (activated) (**Figure 3A**) or four days after (resting) (**Figure 3B**) treatment with 100 nM JWH-133 or 1 µM pertussis toxin and exposure to virus. We found that JWH-133 and pertussis toxin partially inhibited viral replication in both activated and resting cultures (**Figure 3**). Consistent with previous reports [19], inhibition by pertussis toxin was augmented in resting cultures as compared to activated cultures (59.38±29.5% versus 41.16±20.6%, respectively, *p<0.05) (mean±SEM, n = 4). Similarly, we found that the efficacy of JWH-133–mediated viral inhibition was significantly increased in resting, as compared to activated, cultures (63.34±31.7% versus 36.31±18.2%, respectively, *p<0.05 (mean±SEM, n = 4) (**Figure 3C**). These data are consistent with the premise that alteration of GPCR signaling predominantly alters viral infection in resting cells.

### CB_2_R agonism inhibits SDF-1α mediated CXCR4 signaling

**Figure 4:**
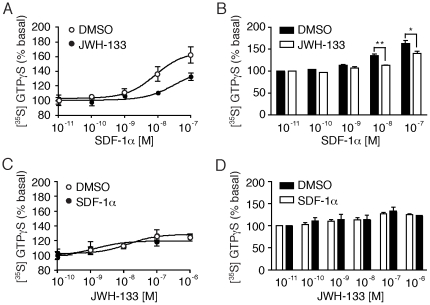
CB_2_ agonism inhibits SDF-1á mediated G-protein coupling to CXCR4. (A–B) GTPãS binding following addition of the CXCR4 agonist SDF-1á to permeabilized primary CD4+ T cells pretreated with either DMSO or 100 nM of the CB_2_ agonist JWH-133 (A) in a representative donor and (B) in multiple donors (mean±SEM; n = 4 donors; **p = 0.0034, *p<0.02). (C–D) GTPãS binding following addition of the CB_2_ agonist JWH-133 to permeabilized CD4+ T cells pretreated with either DMSO or 100 nM of the CXCR4 agonist SDF-1á (C) in a representative donor and (D) in multiple donors (mean±SEM; n = 4 donors).

Our data suggested that CB_2_R activation specifically inhibited CXCR4-tropic virus and that this effect was greatest in resting cells. Given a previous report, which indicated that CXCR4 signaling enhances HIV-1 infection in resting cells [19], we chose to investigate the functional consequence of CB_2_R stimulation on CXCR4-mediated signaling. To assay for CXCR4 activity, we first measured changes in a G protein activity in CD4+ T cells activated by the cytokine SDF-1α (CXCL12). SDF-1α treatment led to a robust, dose-dependent increase in [^35^S]GTPγS binding in CD4+ T cells (**Figure 4A**). Pretreatment with 100 nM JWH-133 significantly decreased this effect at higher doses (16.7±8.37%, **p = 0.0034) (mean±SEM, n = 4 donors) (**Figure 4B**). In contrast, pretreatment with SDF-1α did not inhibit JWH-133 induced [^35^S]GTPγS binding at any concentration tested (**Figure 4C**). CB2 agonism therefore decreases G-protein activation in response to the CXCR4 agonist SDF-1α.

**Figure 5:**
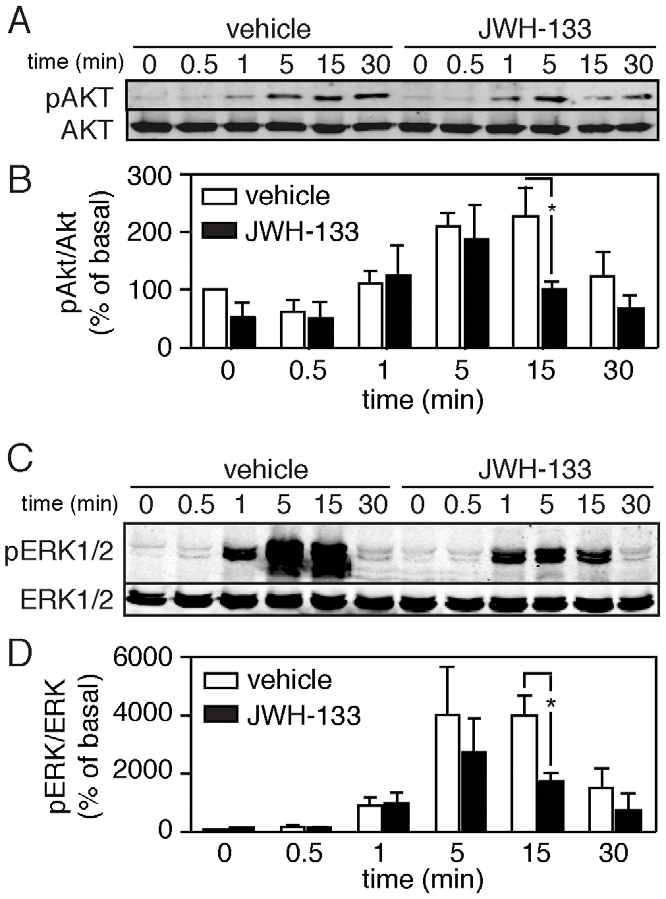
CB_2_ agonism inhibits acute SDF-1á mediated signaling in primary CD4+ T cells. (A–B) SDF-1á induced phosphorylation of Akt and (C–D) p42/44 MAP kinase is downregulated in CD4+ T cells pretreated with 100 nM of the CB2 agonist JWH-133. (A, C) Representative western blots of phospho-kinase expression following SDF-1á treatment for 0, 0.5, 1, 5, 15 or 30 minutes. (B, D) Quantification of kinase phosphorylation in multiple donors (mean±SEM; n = 3 donors; *p<0.05) taken over basal, defined as signal at time 0.

The CB_2_R-induced inhibition of CXCR4 signaling was also indicated by a decrease in phosphorylation of downstream kinases following SDF-1α treatment (**Figure 5**). We assayed SDF-1α-induced Akt and p32/44 MAP kinase (ERK 1/2) phosphorylation in CD4+ T cells following treatment with or without CB_2_R agonist JWH-133. SDF-1α-mediated phosphorylation of both Akt (**Figure 5A–B**) and ERK 1/2 (**Figure 5C–D**) was significantly inhibited by pretreatment with JWH-133. These results suggest that the activation of CB_2_R is sufficient to inhibit downstream CXCR4-mediated signaling pathways.

### The CB_2_R agonist JWH-133 decreases F-actin in CD4+ T cells

**Figure 6:**
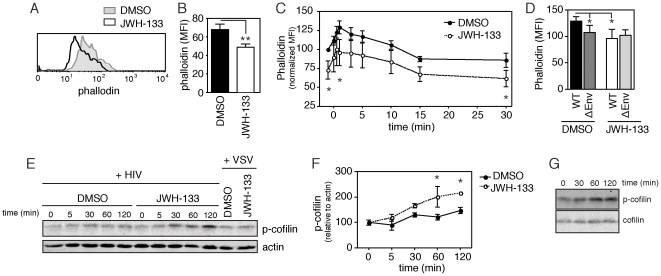
Alteration of actin organization in primary CD4+ T cells pretreated with CB_2_ agonist. (A–B) Total F-actin, as measured by phalloidin binding, following 3 hours of pretreatment with 100 nM of the CB2 agonist JWH-133 in (A) a representative donor and (B) multiple donors (mean±SEM; n = 7 donors; **p = 0.0021). (C) Normalized change in phalloidin binding as a result of HIV-1-mediated CXCR4 activation in CD4+ T cells pretreated with DMSO or JWH-133 (mean±SEM; n = 4 donors; *p<0.05). (D) Mean phalloidin binding in DMSO or JWH-133 pretreated CD4+ T cells treated for 1 minute with wild type (WT) or Env null (ÄEnv) HIV-1 (mean±SEM; n = 4 donors; *p<0.05). (D–E) Cofilin phosphorylation following HIV-1 or VSV-pseudotyped viral exposure in CD4+ T cells pretreated with DMSO or JWH-133 in (D) a representative donor and (E) in multiple donors (mean±SEM; n = 3 donors; *p<0.05). (F) Cofilin phosphorylation and total cofilin following HIV-1 exposure in CD4+ T cells treated with JWH-133.

Down-regulation of CXCR4 signaling with pertussis toxin has been shown to decrease actin dynamics, disrupting the remodeling of the cortical actin barrier required for HIV-1 infection [19]. Given the capacity for CB_2_R agonism to inhibit upstream CXCR4-mediated signaling events, we hypothesized that CB_2_R could suppress CXCR4-induced actin polymerization. To test for this possibility, we assayed the ability of JWH-133 to inhibit SDF-1α mediated actin polymerization, as visualized by phalloidin binding. While a significant difference in the rate of SDF-1α induced F-actin accumulation was not detected (data not shown), we did find that JWH-133-treatment led to a significant decrease in phalloidin binding as compared to untreated control cells (**Figure 6A–B**) (at time 0, 46.2±17.46 versus 64.36±24.34 MFI, respectively; after 30 minutes of SDF-1α treatment, 23.36±11.68 versus 31.3±15.65 MFI, respectively) (mean±SEM, n = 7, **p = 0.0021, *p = 0.04). Although CB_2_R agonism was found to disrupt SDF-1α induced G protein binding and signaling (**Figure 4** **and** **5**), this decrease did not translate into acute disruption of the rate of actin polymerization following SDF-1α treatment. Therefore it is possible that CB_2_R exerts its effect at the level of F-actin formation as treatment with JWH-133 reduces the level of F-actin in the steady state.

During HIV-1 infection, the virus itself acts as an agonist to stimulate CXCR4 and induce actin remodeling in resting cells [46], [47]. We tested to see whether changes in actin reorganization caused by viral binding were altered by CB_2_R agonist pretreatment. To do so, we pretreated CD4+ T cells with JWH-133, incubated these cells with HIV-1 viral particles, and then measured changes in phalloidin binding to F-actin over time. We found that HIV-1 induced transient upregulation in phalloidin binding activity peaking at approximately 1 minute after addition in both control and JWH-133 treated cells (**Figure 6C**). Like with SDF-1α treatment, the rate of increase in phalloidin binding was not significantly altered in CB_2_R agonist pretreated cells. Consistent with our previous observations, we found that that basal phalloidin binding was significantly reduced in JWH-133 treated cells. The reduction in F-actin formation in JWH-133 treated cells was consistent over time despite the transient upregulation of phalloidin stain induced by virus (**Figure 6C**). Taken together, these data suggest that reduction of F-actin induced by CB_2_R agonism results in the reduction of total F-actin over time, despite addition of CXCR4 agonists such as SDF-1α or HIV-1 viral particles. The rate of actin polymerization remained constant after acute CXCR4 activation, but the total amount of F-actin induced was significantly lower in cells pretreated with CB_2_R agonist. Indeed, the amount of F-actin induced by wild type virus in JWH-133 pretreated cells was similar to that induced by Env-null viral particles in control treated cells (at 1 minute, 107.5±53.7 versus 95.9±47.9) (mean±SEM, n = 4) (**Figure 6D**). Therefore, JWH-133 treatment reduces F-actin concentration to background levels, that is, the same level as non-specific induction of actin by Env-null virus. This basal reduction in actin polymerization via CB_2_R may reduce actin rearrangement sufficiently to block viral infection.

### CB_2_R agonism decreases HIV-induced cofilin activation

HIV-triggered actin rearrangement is regulated in part by the severing protein cofilin, which dissociates and facilitates depolymerization of actin filaments thereby promoting actin dynamics [45]. In the inactive state, cofilin is phosphorylated; agonism of Gαi-coupled chemokine receptors initiates cofilin de-phosphorylation and activity [20]. Induction of cofilin de-phosphorylation increases HIV infection [19], we therefore hypothesized that JWH-133 treatment, which decreases HIV infection, would lead to increased cofilin phosphorylation. To identify changes in cofilin regulation, we measured phosphorylated cofilin (p-cofilin) expression in CD4+ T cells pretreated with JWH-133 and exposed to HIV. We detected a significant increase of p-cofilin over time in JWH-133 treated cells, as compared to control treated cells (216.42±124.95 versus 146.83±84.77, respectively, p-cofilin density normalized to actin at 120 minutes) (mean±SEM, n = 3, *p<0.05) (**Figure 6E–F**). This data suggests that CB_2_R agonism not only blocks HIV-induced cofilin de-phosphorylation, but also enhances p-cofilin. Indeed, CB_2_R-induced p-cofilin was also observed in cells exposed to a control, VSV-envelope pseudotyped virus (183.11±91.56 JWH-133 versus 138.32±69.16 control, p-cofilin density normalized to actin at 120 minutes) (mean±SEM, n = 4, *p<0.05) (data not shown). We did not observe changes in total cofilin levels in cells treated with HIV and JWH-133 (Figure 6G). Taken together, our data indicate that CB_2_R agonist pretreatment leads to accumulation of p-cofilin as well as the inhibition of cofilin dephosphorylation, i.e. activation in the presence of HIV; findings consistent with a known requirement for cofilin activation in HIV infection [19]. The increase of inactive cofilin in JHW-133 treated cells is a likely mechanism for the reduction of actin dynamism and subsequent inhibition of viral infection in these cells.

### CB_2_R agonism inhibits productive infection, but not viral fusion

**Figure 7:**
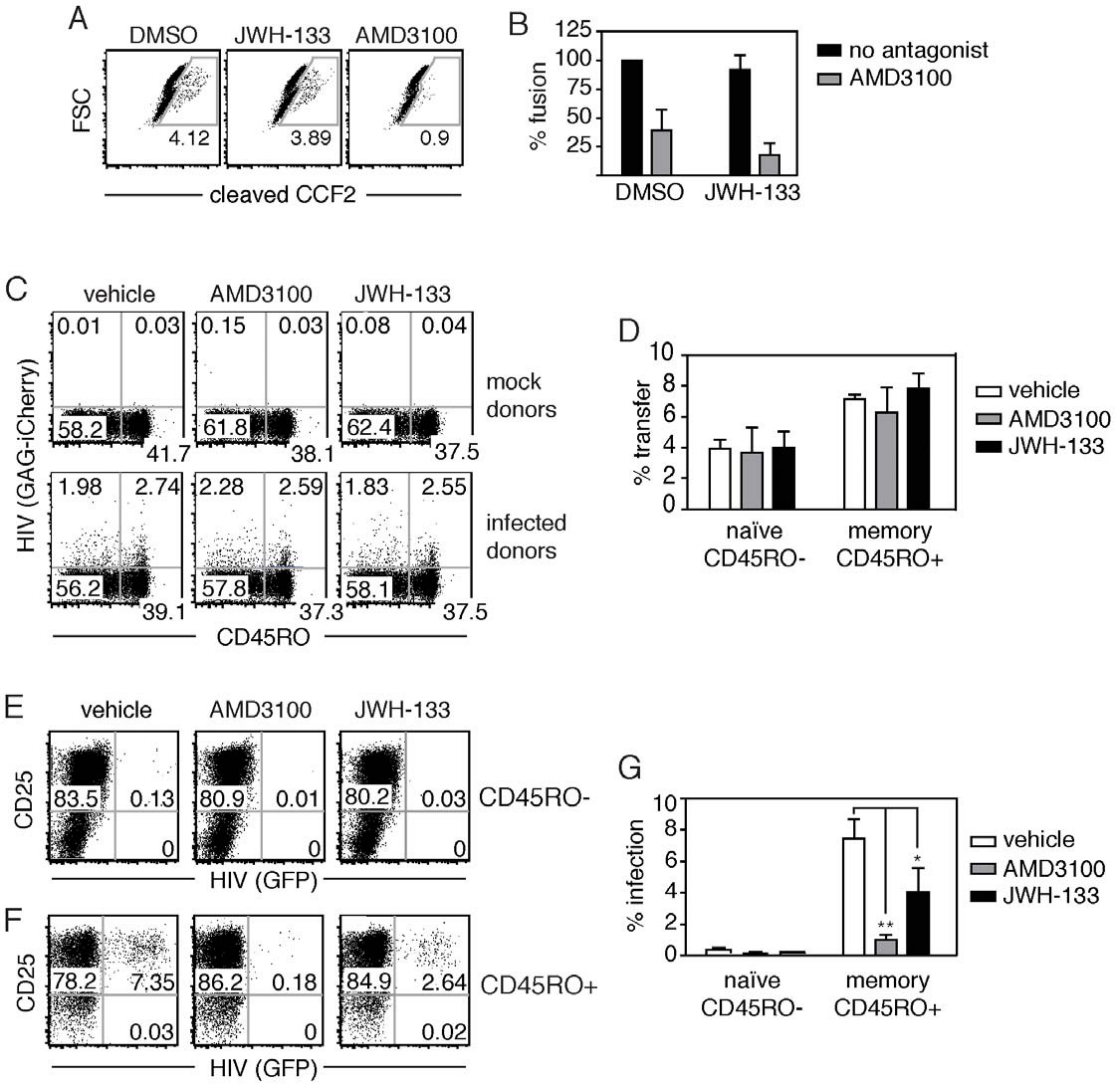
CB_2_ agonism inhibits productive infection of CXCR4-tropic HIV-1, but not cell-associated transfer or viral fusion. (A–B) Viral fusion, as indicated by â-lactamase cleavage, in primary CD4+ T cells pretreated with either JWH-133 or AMD3100 in (A) a representative donor and (B) in multiple donors (mean±SEM; n = 4 donors). (C) Transfer of HIV (Gag-iCherry) from infected Jurkat donors to memory (CD45RO+) or naive (CD45RO-) primary CD4+ T cells pretreated with 100 nM of the CB2 agonist JWH-133 or the CXCR4 antagonist AMD3100, in a representative donor. (D) Quantification of viral HIV (Gag-iCherry) transfer from Jurkat donors after 3 hours in CD45RO+ and CD45RO- cells pretreated with JWH-133 or AMD3100 (mean±SEM; n = 4 donors). (E–G) Productive infection following HIV transfer from primary CD4+ donors infected with HIV (GFP). Pretreated CD4+ targets were cocultured with infected CD4+ donor for 3 hours, sorted by CD45RO expression, and then activated. HIV (GFP) expression is shown in (E) CD45RO- naive or (F) CD45RO+ memory CD4+ T cells after 4 days of activation with IL-2 and anti-CD3/CD28 or anti-CD3 alone, respectively. (G) Quantification of productive infection post-activation in sorted CD45RO- or CD45RO+ CD4+ T cells pretreated with JWH-133 or AMD3100 (mean±SEM; n = 4 donors; **p = 0.0099; *p<0.05).

Alteration of actin dynamics has been shown to inhibit productive viral infection but not viral binding or fusion [46]. To confirm that CB_2_R-mediated changes in the actin cytoskeleton did not inhibit viral fusion, we assessed levels of fusion using a β-lactamase (BlaM)-Vpr fusion assay [37]. NL4.3 virions that incorporated BlaM-VPR were used to infect JWH-133 and control treated cells loaded with the β-lactamase substrate CCF2-AM. Upon viral membrane fusion, BlaM-Vpr is released into the cytoplasm where it is able to cleave CCF2-AM. Infection of primary, resting CD4+ T cells induced fusion in approximately 4% of cells (**Figure 7A**). We found that blockade of HIV binding with the CXCR4 antagonist AMD3100 significantly reduced viral fusion and CCF2-AM cleavage, but pretreatment with JWH-133 had no effect (**Figure 7** **A–B**). This data indicates that CB_2_R antagonism of CXCR4 function does not block the early stages of viral infection binding and fusion.

### CB_2_R agonism inhibits cell-associated viral infection, but not transfer

Our findings suggest that CB_2_R agonism strongly inhibits post-fusion events during viral infection in resting cells exposed to cell-free virus. Given these observations, we wanted to determine the capacity for CB_2_R agonism to block viral transmission and infectivity in a cell-associated model of infection. Cell-associated viral infection is hundreds to thousands-fold more efficient than cell-free infection [33], [48]. During cell-associated transmission, a synaptic structure, called the viral synapse, is formed between the infected (“donor”) and non-infected (“target”) cell [49]. Significant actin rearrangements accompany formation of the virological synapse [50], and these actin structures have been hypothesized to regulate viral penetration into the target cell [51]. Unlike cell-free viral infection, transfer of virus between cells is co-receptor independent; blockade of viral binding to CXCR4 with a selective antagonist, AMD3100, does not inhibit passage of virus [34]. Once virus is captured within a target cell, however, co-receptor binding is still required for viral fusion. We hypothesized that CB_2_R agonism, like the CXCR4 antagonist AMD3100, would not inhibit viral transfer, but would block productive infection.

To assay the impact of CB_2_R agonism on cell-associated viral transfer, we used a CXCR4-tropic NL4-3-based reporter virus called HIV Gag-iGFP, which carries an interdomain insertion of green fluorescent protein (GFP) in the core structural protein Gag [52]. Each mature viral particle contains ∼5000 GFP molecules**^(53)^**, so viral transmission can be measured with high sensitivity [34]. We pretreated CD4+ T cell targets with AMD3100 or JWH-133, and then co-cultured these cells with dye-labeled Jurkat donors infected with the HIV Gag-iCherry reporter virus. After three hours of co-culture, we assessed viral transmission to the target population (**Figure 7C–D**). As previously reported, we found no difference in expression of the HIV Gag-iCherry reporter virus in CD4+ T cells pretreated with AMD3100 as compared to control treated cells. Likewise, pretreatment with the CB2 agonist JWH-133 did not impair viral transfer into target cells (**Figure 7D**). Within this T cell population, transmission to memory (CD45RO+) CD4+ T cells was approximately 50% more efficient than transfer into naive (CD45RO-) cells. Transfer to both T cell subsets was as efficient in AMD3100 and JWH-133 cells as control treated cells. These findings confirm that CB_2_R-mediated inhibition of CXCR4 function does not impair cell-associated HIV-1 transfer to either naive or memory T cells.

We next sought to determine whether CB_2_R agonist pretreatment blocked productive infection following cell-to-cell transfer of virus. We pretreated cells with either AMD3100 or JWH-133 and then conducted a 3-hour transfer experiment using dye-labeled donor CD4+ T cells infected with the NL-GI virus, the same as was used for cell-free assessment of productive infection (**Figure 1**). We then sorted the naïve (CD45RO-) targets from the memory (CD45RO+) target T cell population and activated both subsets to initiate viral replication. We found that in both naive and memory T cells, both AMD3100 and JWH-133 pretreatment inhibited productive viral infection (**Figure 7E–G**). This inhibition was significant in the memory population, with CB_2_R agonist pretreatment resulting in an approximately 50% decrease in infected cells after four days of culture (4.02±2.0% versus 7.47±3.7% control) (mean±SEM, n = 4, *p<0.05) (**Figure 7F**). Although naive cells overall exhibited productive infection at a much lower frequency than memory cells, JWH-133 pretreatment reduced the number of infected cells (0.02±0.01% versus 0.2±0.1%) (mean±SEM, n = 4) (**Figure 7E**). These results indicate that CB_2_R agonism blocks productive viral infection after cell-associated viral exposure, just as it does with cell-free virus. The capacity for CB_2_R agonism to block infection following viral transfer is consistent with our finding that CB_2_R-mediated inhibition infection occurs after binding, and indeed fusion, in a cell-free system (**Figure 7A–B**). Taken together, these results are consistent with the idea that actin rearrangements inhibited by CB_2_R, while not required for cell-associated viral transfer, are required for productive viral infection following cell-associated transfer.

## Discussion

Human immunodeficiency virus type 1, (HIV-1) infection in T cells requires viral binding to two receptors, CD4+ and a chemokine co-receptor, either CXCR4 or CCR5 [54]. These co-receptors are members of the highly conserved family A of G-protein coupled receptors (GPCRs). Absence of co-receptors, or blockade of HIV-1 binding to one of these co-receptors, are both sufficient to abrogate *de novo* viral infection in a target cell [55]. Similarly, manipulation of these GPCRs with pharmacological ligands that alter co-receptor recycling [56], binding pocket occupancy [57], or co-receptor activity [19] also inhibit viral replication.

Here, we report that cannabinoid activation of CB_2_R inhibits CXCR4-tropic HIV infection by altering CD4+ T cell actin dynamics. We find that selective CB_2_ activation blocks both cell-free and cell-associated viral infection, reducing the frequency of infected cells by 30-60% (**Figures 1** **,** **7**). This inhibition is pronounced in resting cells, which are a target of CXCR4-tropic HIV [44]. Additionally, this inhibition is mediated post-transfer during cell-to-cell infection and post-fusion in the target cell following infection with cell-free virus (**Figure 7**). We further investigated the mechanism by which CB_2_R agonism altered HIV permissiveness. Our findings demonstrate that CB_2_R activation at concentrations sufficient to inhibit virus does not alter CXCR4 levels of surface expression, but does significantly reduce CXCR4-mediated G-protein binding and downstream signaling (**Figure 4** **and** **5**). This inhibition of CXCR4 signaling is accompanied by a loss in F-actin accumulation (**Figure 6**), which may prevent the cortical actin rearrangements required for reverse transcription and migration of the viral preintegration complex to the nucleus [58]. Taken together, our results suggest that CB_2_ cross-regulates CXCR4 and that this inhibitory cross-talk is sufficient to decrease viral infection.

Although we here identify cross-talk between the CB_2_R and CXCR4 receptors and downstream impairment of actin dynamics, the possibility remains that CB_2_R activation results in induction of unknown anti-viral host factors. Arguing against the possible induction of unknown anti-viral factors, CB_2_R agonism did not block HIV infection by virus bearing the CCR5-tropic JRFL envelope (**Figure 1C**). The inability of CB_2_ to inhibit CCR5-tropic virus suggests that CB_2_-mediated alterations to the target cell are negligible in the predominantly memory CCR5+ CD4+ T cell subset. This effect is unlikely to be specific to memory cells as a whole, as CB_2_R treatment efficiently blocked productive infection in both memory and naive cell subsets following infection with cell-associated X4-tropic HIV-1 (**Figure 7F–G**). Rather, these findings may indicate that infection with CCR5-tropic virus is less dependent on chemokine-receptor mediated signaling and *de novo* cytoskeletal rearrangement for productive infection.

The CB_2_R may be considered as an adjunct therapeutic target for inhibition of CXCR4-tropic viral spread to resting T cell populations in patients with AIDS. A particularly compelling therapeutic rationale for the evaluation of antiviral affects of CB_2_R agonists may be to address severe symptoms of cachexia or neuropathic pain which may also present in patients with AIDS, without the adverse neurological or behavioral side effects associated with CB_1_R agonism [59]. Although the effect of CB_2_R agonists on HIV infection is moderate, an accumulated effect in patients treated daily for pain could explain positive effects on viral load over time. We find that CB_2_R agonist pretreatment of resting cells inhibits viral spread in a receptor-selective manner in resting cells and does broadly inhibit T cell activation (**Figure 2**). Although previous studies have indicated that pan-cannabinoid agonists can possess an immunosuppressive function *in vitro* [11] and *in vivo* [42], [43], ablation of CB_2_R in mice was not found to increase T cell number, proliferation, or apoptosis in the periphery [14]. Immunosuppression by CB_2_R may be attributed in part to drug toxicity at high concentrations of cannabinoid agonist. Indeed, the use of cannabinoid drugs in patients with HIV is associated with an increase, rather than a decrease, in CD4+ T cell number [1] and has been shown to reduce viral load in SIV infected rhesus macaques [9]. It is possible that novel CB_2_R-specific agonists and allosteric modulators that exert potent anti-viral activity without inducing immunosuppression could be identified. Further study of cannabinoids and other neuroendocrine regulators that selectively modulate immune function may result in the discovery of new anti-viral drugs that can also mitigate AIDS-associated symptoms.
